# Supplementary material for: Insights into the Microstructural Evolution Occurring during Pyrolysis of Metal-Modified Ceramers Studied through Selective SiO2 Removal
Source: Materials (Basel). 2021 Jun 14;14(12):3276. doi: 10.3390/ma14123276 (PMC8231856; doi:10.3390/ma14123276)
Supplement: Supplementary file 1 [file materials-14-03276-s001.zip › materials-1244516-supplementary.pdf]

materials
MDPI

Supplementary Material

Insights into the Microstructural Evolution Occurring during Pyrolysis of Metal-Modified Ceramers Studied through Selective SiO_2 Removal

Aitana Tamayo [1,*], Juan Rubio [1], Fausto Rubio [1] and Mª Angeles Rodriguez [2]

[1] Department. of Physical Chemistry of Surfaces and Processes, Institute of Ceramics and Glass, CSIC. Kelsen 5, 28049 Madrid, Spain; jrubio@icv.csic.es (J.R.); frubio@icv.csic.es (F.R.)
[2] Department. Of Environmental Sciences, Faculty of Sciences, University of Extremadura, Avda. Elvas s/n, 06071 Badajoz, Spain; marodgon@unex.es
* Correspondence: aitanath@icv.csic.es; Tel.: +34-91-735-5840

Citation: Tamayo, A.; Rubio, J.; Rubio, F.; Rodríguez, Mª.A. Insights into the Microstructural Evolution Occurring during Pyrolysis of Metal-Modified Ceramers Studied through Selective SiO_2 Removal. *Materials* **2021**, *14*, x. https://doi.org/10.3390/xxxxx

Academic Editor(s): Juergen Stampfl

Received: 17 May 2021
Accepted: 11 June 2021
Published:14 June 2021

Publisher's Note: MDPI stays neutral with regard to jurisdictional claims in published maps and institutional affiliations.

Copyright: © 2021 by the authors. Submitted for possible open access publication under the terms and conditions of the Creative Commons Attribution (CC BY) license (http://creativecommons.org/licenses/by/4.0/).

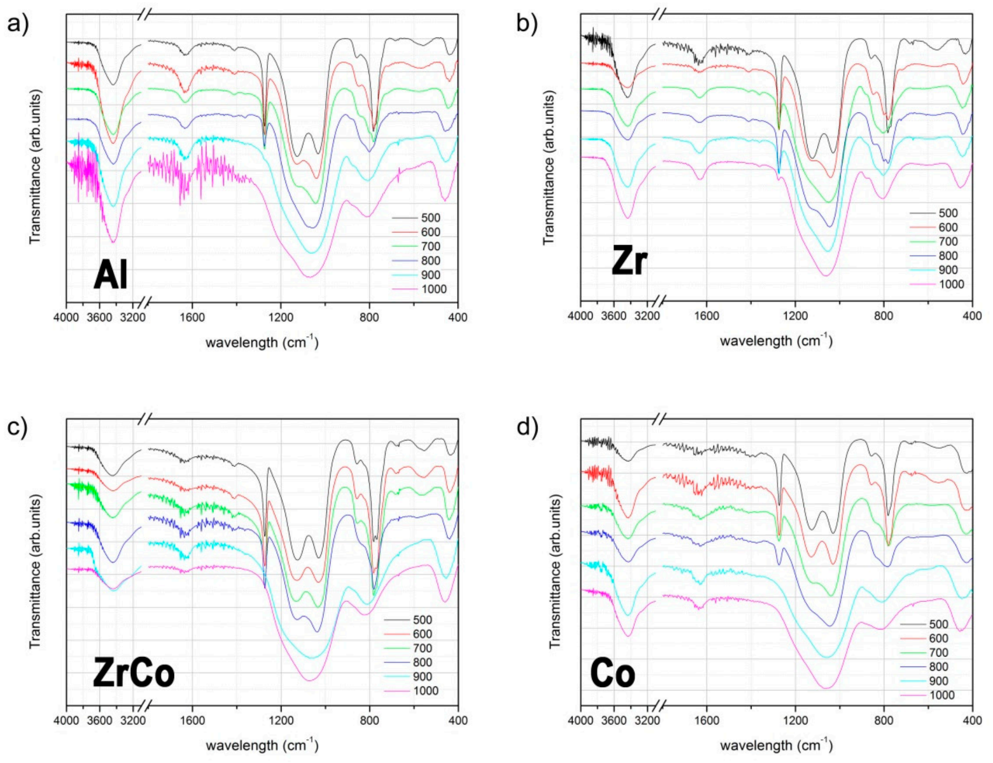

Figure S1. Infrared spectra of the samples pyrolyzed at ifferent temperatures and containing the heteroatoms: (**a**) Aluminum; (**b**) Zirconium; (**c)** Zirconium plus Cobalt; (**d**) Cobalt.

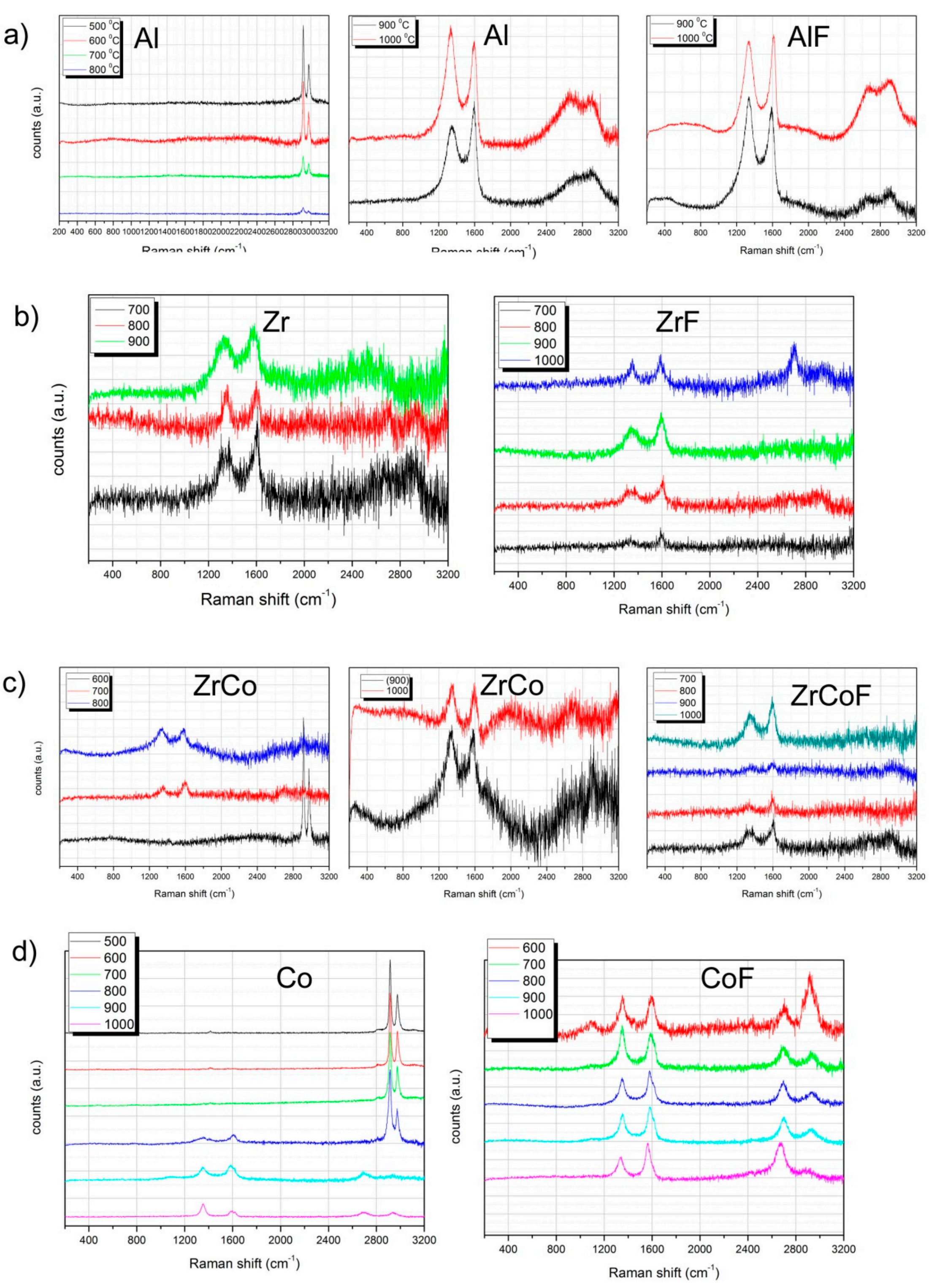

Figure S2. Raman spectra of the pyrolyzed and HF-etched samples and containing the heteroatoms: (**a**) Aluminum; (**b**) Zirconium; (**c**) Zirconium plus Cobalt; (**d**) Cobalt.

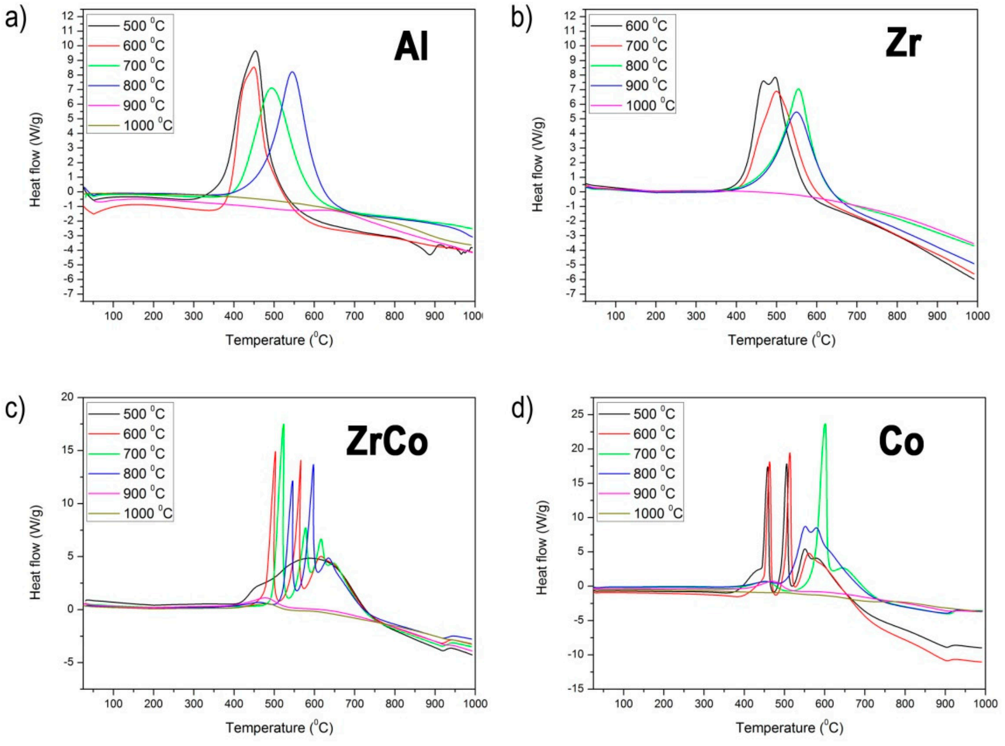

Figure S3. Differential scanning calorimetry curves of the pyrolyzed materials and containing the heteroatoms: (**a**) Aluminum; (**b**) Zirconium; (**c)** Zirconium plus Cobalt; (**d**) Cobalt.

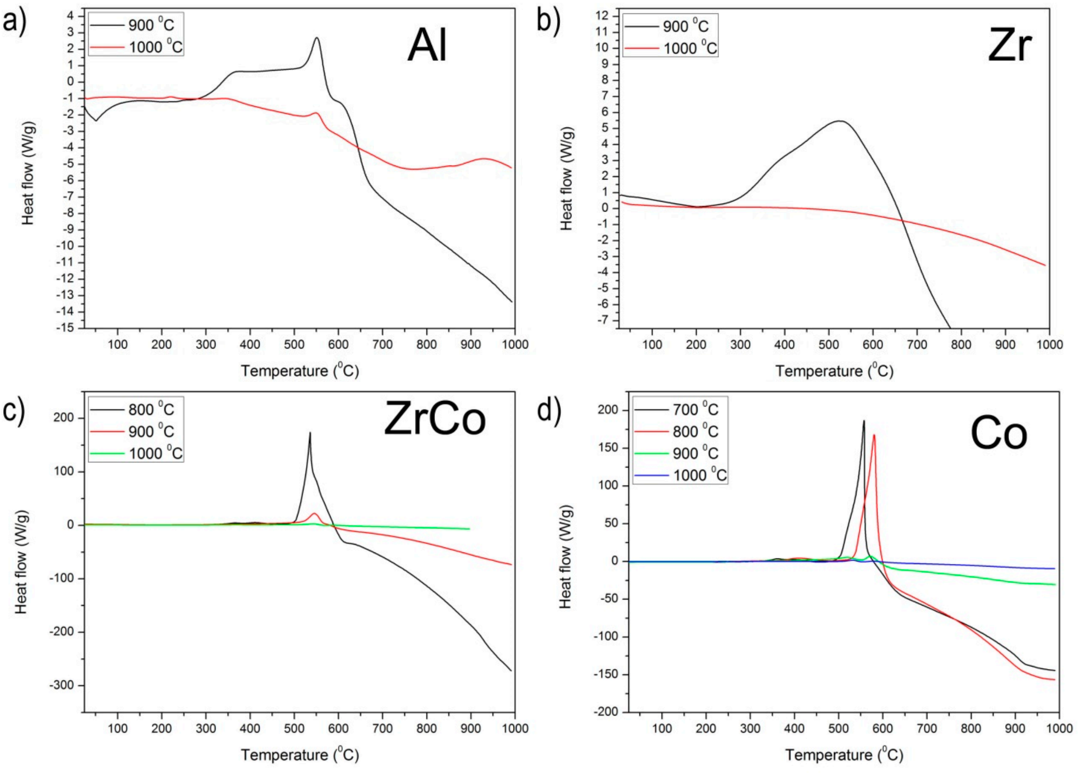

Figure S4. Differential scanning calorimetry curves of the HF-etched samples and containing the heteroatoms: (**a**) Aluminum; (**b**) Zirconium; (**c)** Zirconium plus Cobalt; (**d**) Cobalt.

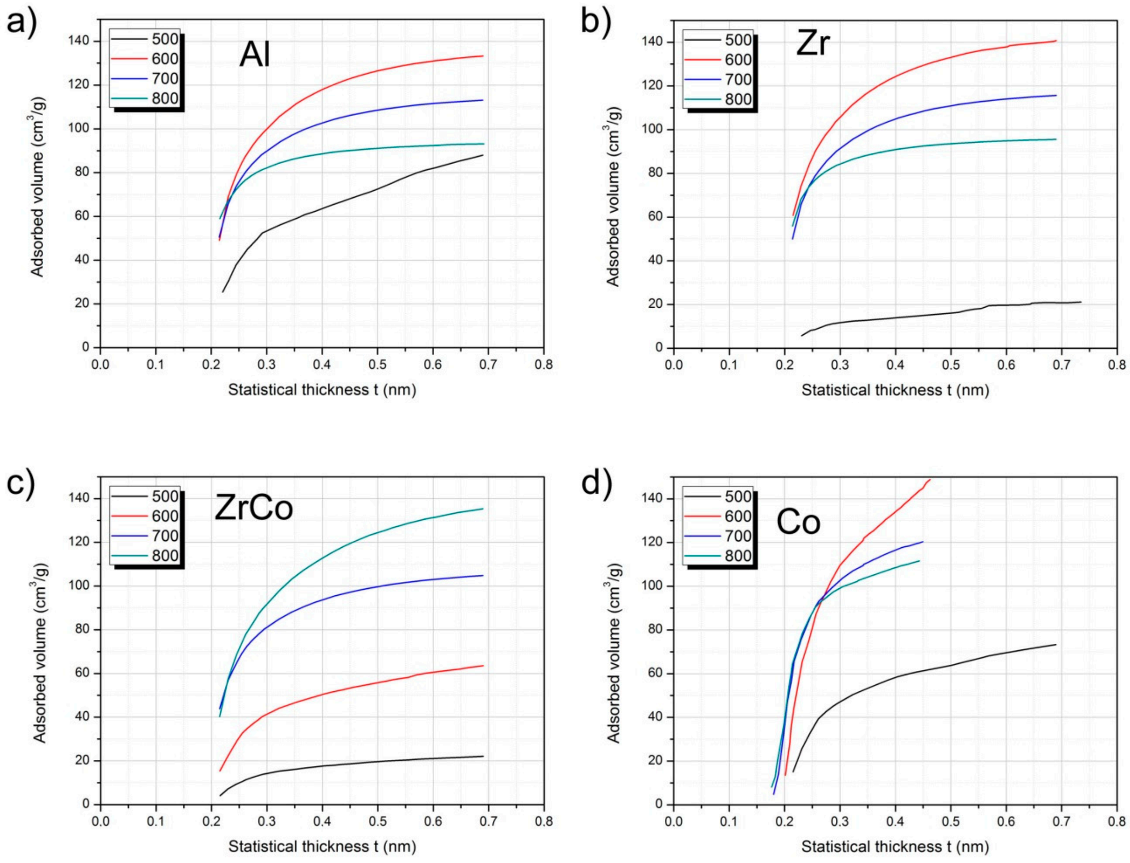

Figure S5. t-plots obtained from the nitrogen adsorption isotherms of the pyrolyzed materials and containing the heteroatoms: (**a**) Aluminum; (**b**) Zirconium; (**c**) Zirconium plus Cobalt; (**d**) Cobalt.

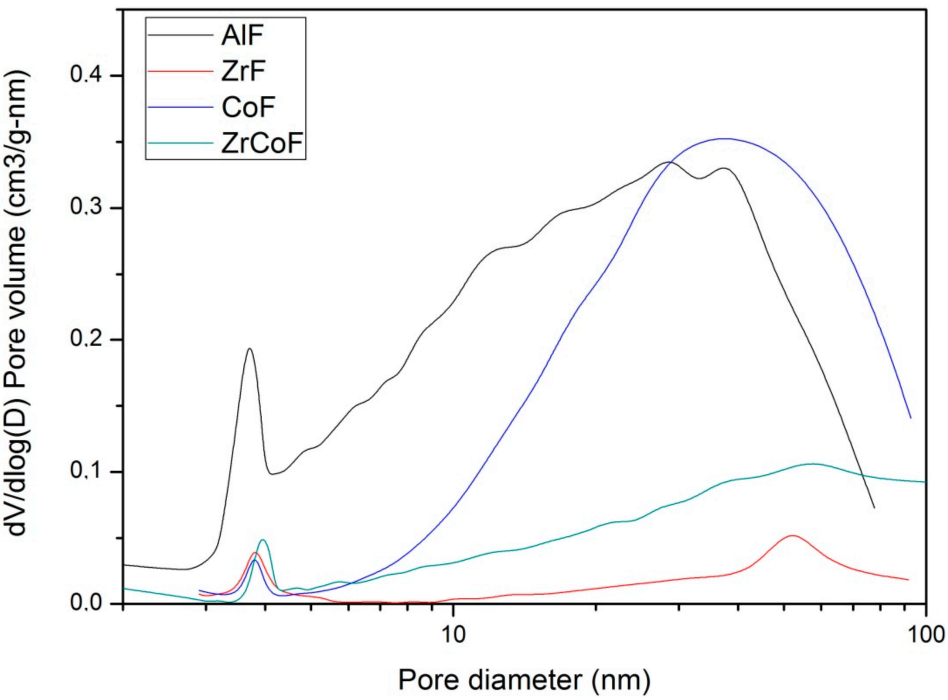

Figure S6. BJH Pore size distributions of the HF-etched samples.

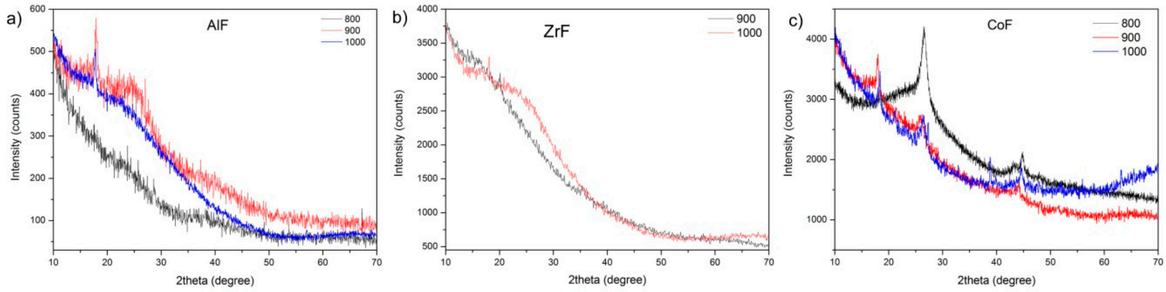

Figure S7. XRD patterns of the HF-etched materials and containing the heteroatoms: **a**) Aluminum; (**b**) Zirconium; (**c**) Cobalt.
